# Supplementary material for: Modelling Skylarks (Alauda arvensis) to Predict Impacts of Changes in Land Management and Policy: Development and Testing of an Agent-Based Model
Source: PLoS One. 2013 Jun 6;8(6):e65803. doi: 10.1371/journal.pone.0065803 (PMC3675089; doi:10.1371/journal.pone.0065803)
Supplement: Supporting Information S4 — The skylark ODdox as a zipped archive. (ZIP) [file pone.0065803.s004.zip › Skylark_ODdox/class_low_priority.html]

ALMaSS Skylark ODdox: LowPriority< ELEMTYPE > Class Template Reference


ALMaSS Skylark ODdox
 2.0


- Main Page
- Related Pages
- Classes
- Files

- Class List
- Class Index
- Class Hierarchy
- Class Members

Public Member Functions

LowPriority< ELEMTYPE > Class Template Reference

Used in event handling.
More...

`#include <lowqueue.h>`

List of all members.

|  |  |
| --- | --- |
| Public Member Functions | |
| LowPriPair< ELEMTYPE > | Bottom (void) |
| bool | Empty (void) |
|  | LowPriority () |
| void | Pop () |
| void | Push (ELEMTYPE a\_element, long a\_priority) |

---

## Detailed Description

### template<class ELEMTYPE> class LowPriority< ELEMTYPE >

Used in event handling.

---

## Constructor & Destructor Documentation

template<class ELEMTYPE>

|  |  |  |  |  |
| --- | --- | --- | --- | --- |
| LowPriority< ELEMTYPE >::LowPriority | ( |  | ) |  |

inline

: priority\_queue <LowPriPair< ELEMTYPE> >()

{}

---

## Member Function Documentation

template<class ELEMTYPE>

|  |  |  |  |  |  |
| --- | --- | --- | --- | --- | --- |
| LowPriPair<ELEMTYPE> LowPriority< ELEMTYPE >::Bottom | ( | void |  | ) |  |

inline

{

return this->top();

}

template<class ELEMTYPE>

|  |  |  |  |  |  |
| --- | --- | --- | --- | --- | --- |
| bool LowPriority< ELEMTYPE >::Empty | ( | void |  | ) |  |

inline

{

return this->empty();

}

template<class ELEMTYPE>

|  |  |  |  |  |
| --- | --- | --- | --- | --- |
| void LowPriority< ELEMTYPE >::Pop | ( |  | ) |  |

inline

{

this->pop();

}

template<class ELEMTYPE>

|  |  |  |  |
| --- | --- | --- | --- |
| void LowPriority< ELEMTYPE >::Push | ( | ELEMTYPE | *a\_element*, |
|  |  | long | *a\_priority* |
|  | ) |  |  |

inline

{

push( LowPriPair<ELEMTYPE>( a\_element, a\_priority ));

}

---

The documentation for this class was generated from the following file:

- lowqueue.h


- LowPriority
- Generated on Thu Jan 10 2013 13:15:36 for ALMaSS Skylark ODdox by
   1.8.1.1
